# Efficient phosphate removal from water using ductile cast iron waste: a response surface methodology approach

**DOI:** 10.3389/fchem.2024.1458420

**Published:** 2024-10-02

**Authors:** Mai Hassan Roushdy, Nada Amr Elkhashab, Ahmed Ibrahim Osman, Dalia Amer Ali

**Affiliations:** ^1^ Chemical Engineering Department, Faculty of Engineering, The British University in Egypt (BUE), El-Sherouk, Cairo, Egypt; ^2^ School of Chemistry and Chemical Engineering, Queen’s University Belfast, Belfast, Northern Ireland, United Kingdom

**Keywords:** adsorption, solid waste, phosphates, thermodynamics, optimization, response surface methodology

## Abstract

Water scarcity is a critical issue worldwide. This study explores a novel method for addressing this issue by using ductile cast iron (DCI) solid waste as an adsorbent for phosphate ions, supporting the circular economy in water remediation. The solid waste was characterized using XRD, XRF, FTIR, and particle size distribution. Wastewater samples of different phosphate ion concentrations are prepared, and the solid waste is used as an adsorbent to adsorb phosphate ions using different adsorbent doses and process time. The removal percentage is attained through spectrophotometer analysis and experimental results are optimized to get the optimum conditions using Design Expert V13. The pseudo-second order (PSO) kinetics model and Langmuir isotherm were fitted with the experimental results with maximum adsorption capacity (q_max_ = 0.28 mg/g). The thermodynamic analysis indicated that this adsorption process was spontaneous based on the negative value of Gibbs free energy (∆G). Additionally, the positive values of enthalpy (∆H) indicated the endothermic nature of this adsorption system. It was able to reach the highest adsorption percentage of 98.9 (%) for phosphate ions from aqueous solutions using response surface methodology (RSM) with optimum conditions of 10 mg/L phosphate ion concentration, pH = 8, normal room temperature, 9 min adsorption, and 0.5 g/L adsorbent dosage.

## 1 Introduction

Water resource scarcity is a significant issue, promoting nations to implement policies that ban industrial and domestic discharge while advocating for wastewater treatment and reuse. Water reuse refers to the utilization of treated wastewater for constructive purposes such as irrigation and industrial processes. The re-usage of water offers an opportunity for augmenting water supplies and terminating the loop between water supply and wastewater disposal, which highlights the significance of the topic mentioned above (Alotaibi et al., 2023). Phosphorus is an essential constituent of living organisms that is usually present in surface waters to a certain degree in various compounds (Mekonnen et al., 2020). The concentration of phosphorus in water at natural conditions is usually balanced, meaning that the present amount is close to the ecological system requirement (Usman et al., 2022a). High levels of phosphorous are considered a problem as they can increase the growth of algae and large aquatic plants, leading to a decrease in dissolved oxygen levels, commonly known as eutrophication, which would subsequently affect aquatic life (Akinnawo, 2023). In addition, excessive intake of phosphorous in drinking water could cause stomach cancer (Abdelhay et al., 2018).

Consequently, strict regulations have been set to decrease the permissible level of phosphorous in wastewater, and thus, various methods were investigated for wastewater treatment to abide by these regulations (Monballiu et al., 2020). For instance, biological treatment is a highly significant method primarily due to reduced expenses (Sakamoto et al., 2020). However, the produced effluent usually does not satisfy the previously mentioned regulations, and thus, it requires further treatment before discharge (Sakamoto et al., 2020). Another approach employed for the removal of phosphate is chemical precipitation, which is considered highly efficient but is hindered by the large amounts of sludge it produces, which usually triggers secondary pollution (Zheng et al., 2023). On the other hand, if the adsorption process is taken into consideration, it would be concluded that it holds the upper hand over the biological and chemical approaches as it does not suffer from limitations (Zheng et al., 2023).

Furthermore, adsorption is one of the most crucial and commonly utilized methods, and hence, finding an adsorbent that is readily available and efficient is the key to attaining a promising method (Wu et al., 2024). Many researchers apply adsorption techniques to adsorb various pollutants, including not only water pollutants (Ahmed et al., 2024; Mansour et al., 2022; Usman et al., 2022b; Brontowiyono et al., 2022; Pourrahim et al., 2020; Pourrahim et al., 2020) but also gas pollutants, using synthetic or solid waste adsorbents (Zhang et al., 2024; Dziejarski et al., 2024; Serafin et al., 2024; Fonseca-Bermúdez et al., 2024; Yu et al., 2024). This leads to the proposed theory of employing solid waste as an adsorbent for phosphate ions as it satisfies not only the requisite but also applies waste management (Wu et al., 2024). Countless studies have been established that address this perspective. A study investigated the employment of electric arc furnace dust for phosphate ion removal using an adsorption technique and concluded a maximum removal percentage of 92.38% at conditions of pH = 7, 1 mg/L as an initial concentration for phosphate ion, 90 min contact time, and 6.5 g/L as an adsorbent dosage (Ali et al., 2021). Another study analyzed three industrial wastes—ferric–alum water treatment residual (FAR), fly ash, and red mud—as adsorbents for phosphate ions, comparing their adsorption capacity with that of two natural adsorbents, namely, zeolite and diatomite (Wang Y. et al., 2016). It concluded the effective utilization of these solid wastes and that their adsorptive capacity was much higher than that of natural adsorbents, which could have originated from their high content of Fe, Al, and Ca (Wang Y. et al., 2016). This is an indication that employing solid waste would be highly prosperous. Ductile cast iron (DCI) is a ferrous alloy that mainly consists of carbon and silicon along with other elements, which are usually controlled to attain various grades and influence other mechanical properties, machinability, and castability. In this research, DCI solid waste was used as an adsorbent for adsorption of phosphate ions from aqueous solutions.

## 2 Materials and methods

### 2.1 Chemicals

DCI solid waste was sourced from Greater Cairo Foundries in Egypt. Analytical-grade reagents, such as concentrated sulfuric acid (H_2_SO_4_ 96%), ammonium molybdate (NH_4_)_6_Mo_7_O_24_, ascorbic acid (C_6_H_8_O_6_), and sodium phosphate monobasic (NaH_2_PO_4_), were all utilized in this investigation. Water that had been double-distilled was used to prepare each solution.

### 2.2 Characterization of the solid waste resulting from the DCI industry

Important analysis techniques were used to characterize the used solid waste as follows:

#### 2.2.1 X-ray diffraction

The apparatus used was an Empyrean diffractometer (Malvern Panalytical, Netherlands). X-ray diffraction (XRD) was used to investigate the degree of purity and crystallinity of the DCI solid waste (Mohamed et al., 2024). Samples were subjected to an XRD examination, spanning a 2θ range of 5.009^o^–99.987^o^.

#### 2.2.2 X-ray fluorescence

American Society for Testing and Materials (ASTM C114-18) was used to determine the solid waste oxide contents (Gong et al., 2023).

#### 2.2.3 Fourier–transform infrared (FTIR)

FTIR determined the surface functional groups for the solid waste (Mohamed et al., 2024) (Vertex 70 RAM II, Germany).

#### 2.2.4 Particle size distribution

Particle size distribution (PSD) was determined using sieving screens set to obey the standard ASTM E 11 /2009 and ASTM D 422/2007.

### 2.3 Water treatment using the adsorption technique

The influencing factors were investigated in batch experiments to assess the effectiveness of the DCI solid waste in phosphorus. The initial concentration of phosphorus (A) was used in the range of 1–10 mg/L. This range was selected following law 4 from the Egyptian Environmental Laws of 1994’s maximum limitations for wastewater discharge into the Nile River (Law Number four, 1994). Contact time (B) was used in the range of 6–90 min. Adsorbent amount (C) was used in the range of 0.5–6.5 g/L. The selection of both contact time and adsorbent amount ranges was based on previous works related to phosphate ion removal using the adsorption technique.

Phosphate ion removal was performed at a pH of 8, a temperature of 25°C, and with vigorous shaking at 180 rpm. After the removal of the ions, the adsorbent was centrifuged to separate it from the water, and then the resulting water samples after treatment were tested using a UV/VIS spectrophotometer to determine the phosphate ion concentration before and after adsorption, thereby assessing the removal efficiency. Equation 1 was used to compute the phosphate ion removal efficiency (Ali et al., 2021):

$$Phosphate\;ions\;removal,\%=\frac{P_{o}-P_{f}}{P_{o}}x10,$$ (1)

where P_o_ and P_f_ are the initial and final ion concentrations of phosphate, respectively.

## 3 Results and discussion

### 3.1 Characterization of the DCI solid waste

#### 3.1.1 X-ray fluorescence

The X-ray fluorescence (XRF) results in Table 1 indicate an 88% composition of MgO, which is attributed to the high melting temperature of cast iron, resulting in the elimination of magnesium. The loss of ignition (L.O.I.%) was because of carbonate-contaminated zinc and iron.

**TABLE 1:** Chemical analysis (XRF) of the used solid waste.

Oxide	MgO	ZnO	Fe_2_O_3_	Na_2_O	SiO_2_	CaO	MnO	TiO_2_	K_2_O	P_2_O_2_	L.O.I
Percentage (%)	88	4.2	2.28	0.4	0.2	0.24	0.04	0.02	0.01	0.01	4.54

#### 3.1.2 Particle size distribution of DCI

**FIGURE 1:**
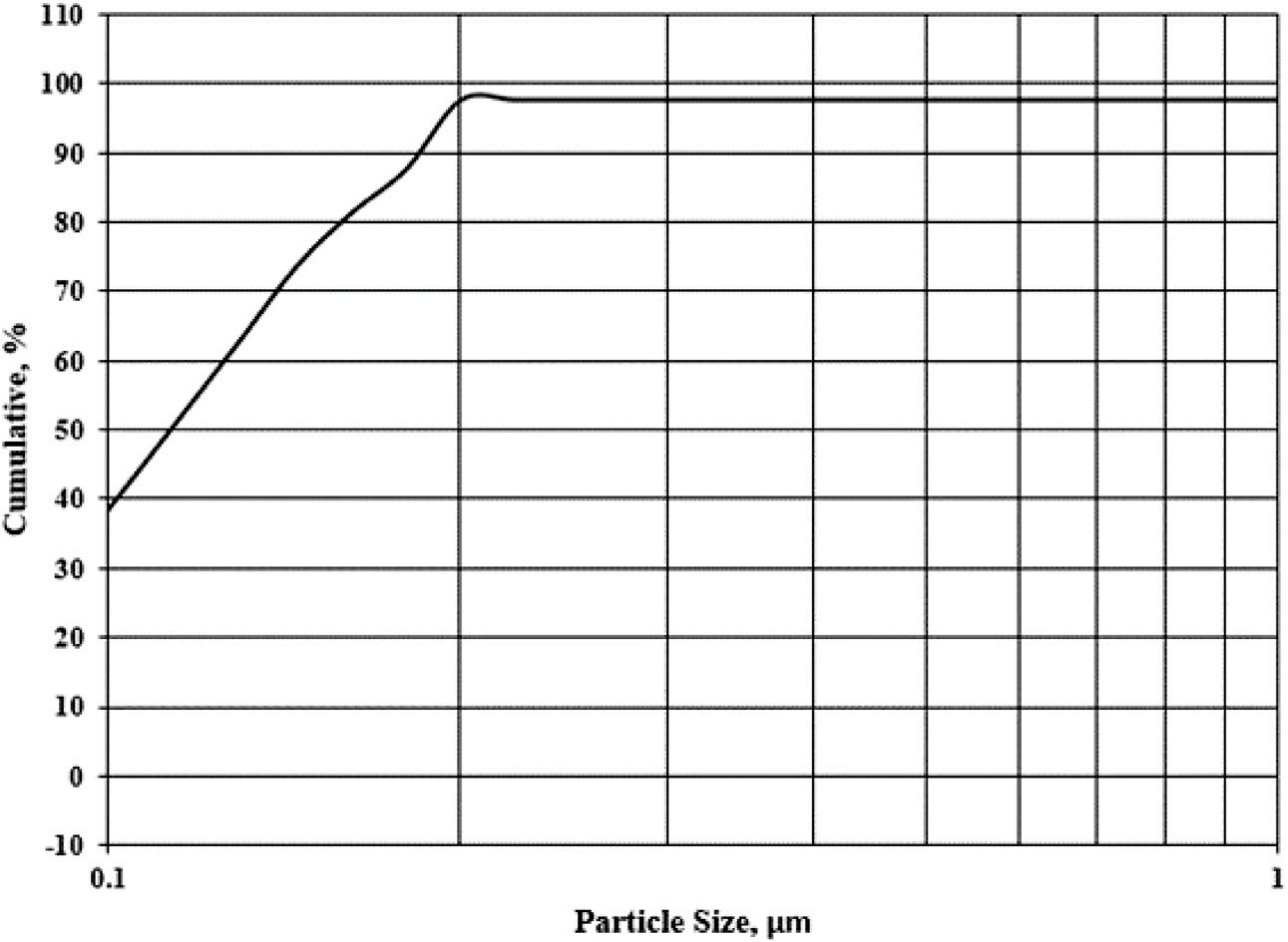
Particle size distribution of DCI solid waste.

Figure 1 shows the DCI solid waste’s cumulative screen analysis curve. The average size of the particles was found to be 0.098 μm. Given the large surface area and multiple active centers, this waste predicted that it would be quite active.

#### 3.1.3 X-ray diffraction

**FIGURE 2:**
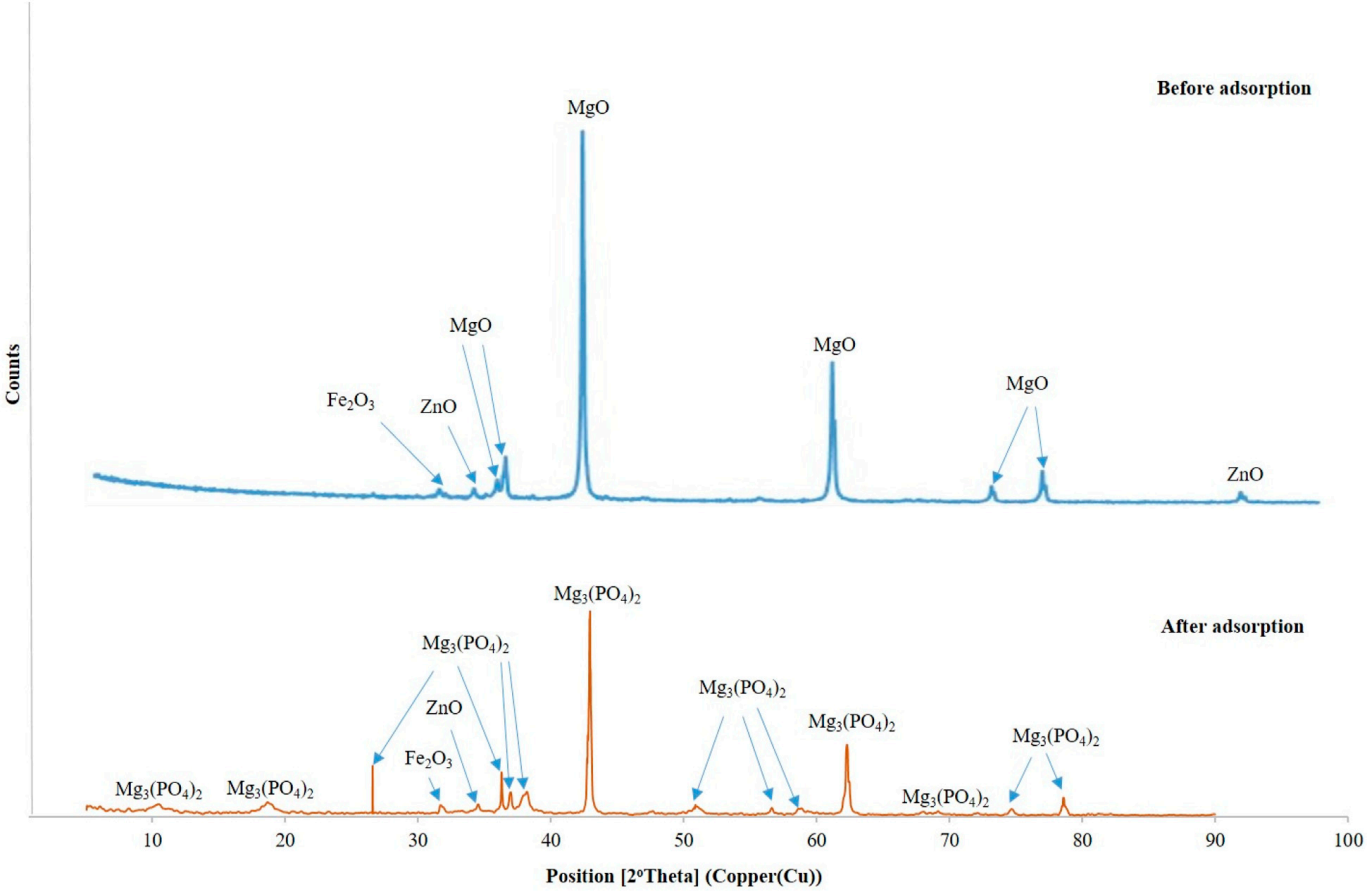
X-ray diffraction results of the solid waste sample.

According to Figure 2, the predominant phase found in DCI solid waste before adsorption of phosphate ions was periclase or magnesium oxide (MgO) in a cubic form at 2Ꝋ = 36.7^o^, 37.04^o^, 42.89^o^, 62.23^o^, 74.8^o^, and 78.6^o^ (Durdu et al., 2011; Nga et al., 2020). Moreover, there were minor phases presented in the DCI solid waste, including zincite (ZnO) at 2Ꝋ = 34.6^o^ and 94.1^o^ (Handore et al., 2014; Luo et al., 2015) and iron oxide (Fe_2_O_3_) at 2Ꝋ = 32^o^ (Suresh et al., 2016; Qayoom et al., 2020).

Figure 2 shows the phases present in the solid waste before and after adsorption, demonstrating the successful adsorption of phosphate ions onto the DCI. This is evidenced by the decrease in intensities of MgO peaks, a slight shift to the right on the *x*-axis, and the conversion of MgO to magnesium phosphate Mg_3_(PO_4_)_2_. The diffraction peaks of Mg_3_(PO_4_)_2_ appeared at 2Ꝋ = 26.55^o^, 10.73^o^, 18.95^o^, 36.25^o^, 37^o^, 38.25^o^, 42.89^o^, 50.85^o^, 56.67^o^, 59.27^o^, 62.31^o^, 69.27^o^, 74.87^o^, and 78.63^o^ (Durdu et al., 2011; Zhang et al., 2017). According to the XRD and XRF results, it was observed that the DCI solid waste contained mainly MgO at 88%. Therefore, the phosphate ion (PO_4_)^3-^ adsorption mechanism depended mainly on MgO, and it could be clarified through the following Equations 2, 3:

$$\text{MgO}+H_{2}O˗\text{ Mg}{\left(\text{OH}\right)}_{2},$$ (2)

$$\text{Mg}{\left(\text{OH}\right)}_{2}+{\text{PO}}_{4}^{3-}\;˗{\text{Mg}}_{3}{\left({\text{PO}}_{4}\right)}_{2}+H_{2}O.$$ (3)

#### 3.1.4 Fourier-transform infrared

**FIGURE 3:**
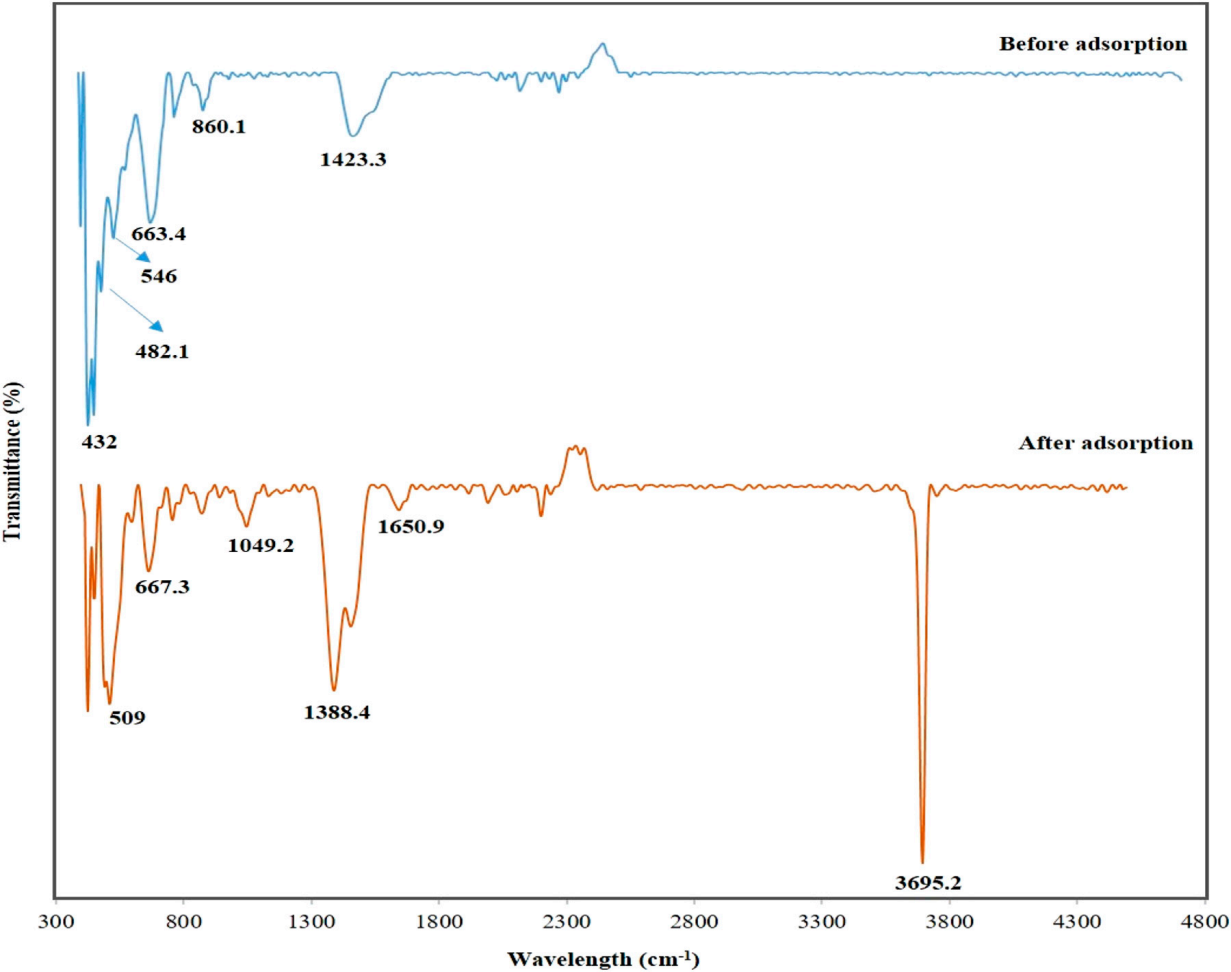
DCI solid waste’s FTIR bands before and after phosphorous adsorption from aqueous solutions.

Figure 3 shows the FTIR pattern for the used adsorbent, DCI solid waste, before and after the adsorption process from its aqueous solution and after. The absorption bands at 432, 663.4, and 860.1 cm^−1^ were attributed to MgO (Zahir et al., 2019; Nga et al., 2020). ZnO was responsible for the absorption bands at 482.1 and 546 cm^−1^ (Handore et al., 2014; Alamdari et al., 2020); before adsorption, the absorption bands at 1,388.4 and 1,423.3 cm^−1^ were attributed to the carboxylate (O–C = O) symmetric and asymmetric stretching vibrations, which were attributed to the carbonate-contaminated zinc and iron (Zahir et al., 2019; Nga et al., 2020).

After the adsorption of phosphate ions, the absorption bands at 1,650.9 cm^−1^ corresponded to the (−OH) group (Zahir et al., 2019). The appearance of the sharp absorption band at 3,695.2 cm^−1^ corresponded to the (−OH) group in Mg(OH)_2_ as when exposed to the atmosphere, MgO surfaces easily absorbed molecules of H_2_O, forming Mg(OH)_2_ (Zahir et al., 2019), which adsorbed phosphate ions from the aqueous solution, resulting in the formation of Mg_3_(PO_4_)_2_.

The appearance of a new absorption band was observed after adsorption at 1,049.2 cm^−1^ corresponding to the phosphorous group (P = O) (El Bouraie and Masoud, 2017; Stuart, 2005), proving the adsorption ability of the DCI solid waste. Furthermore, shifts and decreases in the bands’ intensities of MgO and ZnO after the adsorption process proved that the phosphate ions were successfully adsorbed onto the DCI solid waste surface. Therefore, the FTIR results agreed with the XRD and XRF results.

### 3.2 Adsorption kinetics study

The kinetics study for this adsorption system was performed according to the literature (Usman et al., 2022c; El-Maghrabi et al., 2022; Aziam et al., 2024) to understand the adsorption mechanism. Under the following experimental conditions, this kinetics study was performed under different adsorption times ranging from 6–119 min, pH = 8, 5.5 mg/L phosphate ion initial concentration, 3.5 g/L adsorbent dosage, and normal room temperature. Table 2 shows the results of the kinematics study. These results indicated that the pseudo-second-order best fits the experimental data for phosphorus adsorption using DCI solid waste due to the higher value of R^2^ (0.9985) and the smaller difference between the calculated adsorption capacity (q_e-calculated_) and the experimental adsorption capacity. This conclusion is based on fitting the experimental data to pseudo-first-order and pseudo-second-order kinetic models using Equations 4, 5. The suitability of the pseudo-second-order kinetic model for the adsorption of phosphate ions onto the DCI solid waste suggested that the adsorption process occurred through multiple mechanisms, which included chemical interactions and the electrostatic attraction between the negative phosphate ions and the reactive binding sites on the surface of the DCI solid waste.

**TABLE 2:** Results of the kinetics study.

Adsorption kinetic model	Parameter	Value
PFO “pseudo-first-order reaction kinetics”	qe-experimental (mg/g)	0.281
	qe-calculated (mg/g)	0.071
	K_1_ (min^−1^)	0.0195
	R^2^	0.767
PSO “pseudo-second-order reaction kinetics”	qe-experimental (mg/g)	0.281
	qe-calculated (mg/g)	0.292
	K_2_ (mg/g min)	0.0062
	R^2^	0.9985
Elovich	α (mg/g.min)	0.144
	β (g/mg)	19.72
	R^2^	0.9704

The Elovich model is a notable kinetic model widely used to characterize adsorption systems influenced by chemical properties. This model is applicable in scenarios where the adsorption process involves chemisorption on the surface of the adsorbent, and it is characterized by a decrease in the rate of adsorption over time as the adsorbent surface becomes increasingly covered by the adsorbate (Aziam et al., 2024). The favorable alignment of the experimental data with the Elovich model, as in Equation 6, indicated that a chemisorption mechanism governed this adsorption process. The following are the kinetic models.

• Pseudo-first-order reaction kinetics model

$$\ln\left(q_{e}-q_{t}\right)=-k_{1}t+\ln q_{e}.$$ (4)

• Pseudo-second-order reaction kinetics model

$$\frac{t}{q_{t}}=\left(\frac{1}{q_{e}}\right)\;t+\frac{1}{k\;{q_{e}}^{2}},$$ (5)

where (q_t_) is the ratio between the adsorbed and adsorbent masses at time t and k is the rate constant.

• Elovich kinetic model

$$q_{t}=\frac{\ln\left(\alpha \beta \right)}{\beta }+\frac{1}{\beta }\mathrm{Ln}\left(t\right),$$ (6)

where α is the initial adsorption rate (mg/g/min) and β is the desorption constant associated with the extent of the surface coverage and chemisorption activation energy (g/mg).

**FIGURE 4:**
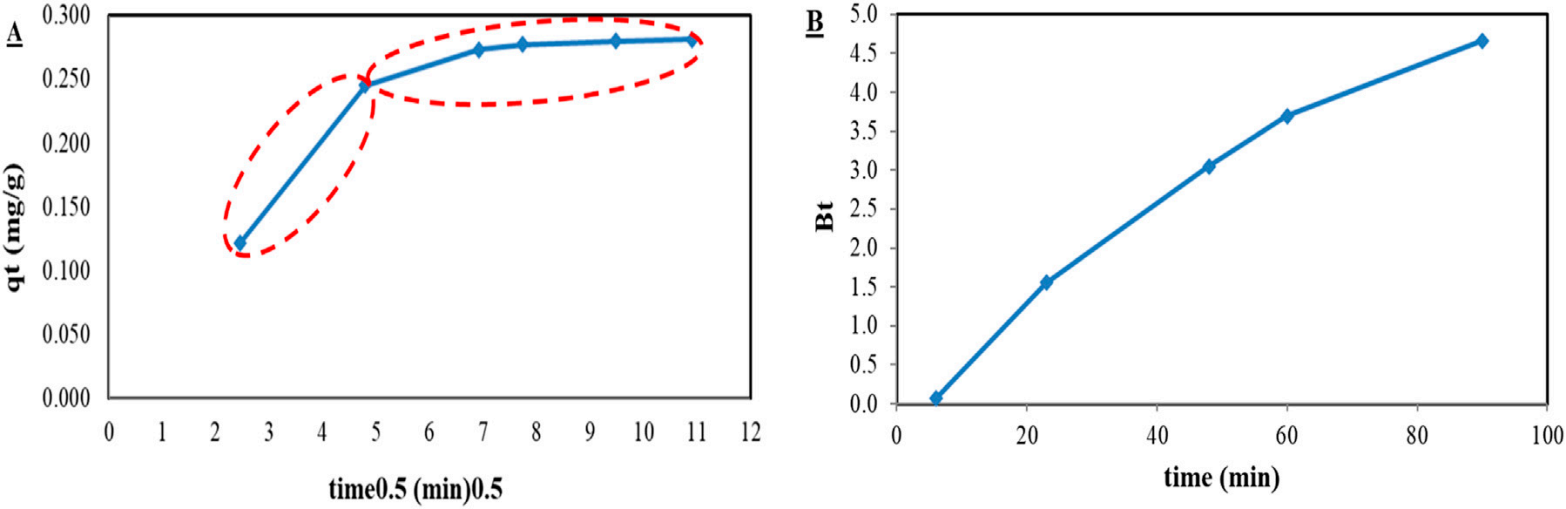
**(A)** Intra-particle diffusion model of phosphate ion adsorption using DCI solid waste, and **(B)** Byod plot of phosphate ion adsorption using DCI solid waste.

The adsorption rate-limiting step was determined using the model of intra-particle diffusion and Byod plot following the guidelines provided in the literature (Wu et al., 2009; Aziam et al., 2024; Nethaji et al., 2013) using Equations 7, 8. Figure 4A represents the intra-particle diffusion model plot, composed of two linear portions; the first portion represents the film diffusion mass transfer step, and the second portion represents the intra-particle diffusion mass transfer step. These results are consistent with the literature, which indicates that the adsorption mechanism, whether physical or chemisorption, relates to the rate-determining step of the adsorption process (Tong et al., 2019; Silva and Baltrusaitis, 2020). If the rate-determining step/the slowest step is film diffusion, the adsorption mechanism would be chemisorption (Tong et al., 2019; Silva and Baltrusaitis, 2020).

The film diffusion was the rate-determining step, as shown in Figure 4B; the Byod plot indicated that it was almost linear and did not pass through the origin (Nethaji et al., 2013). This indicated that the adsorption mechanism was chemisorption involving shared electron pairs via covalent or ionic bonds.

• Intra-particle diffusion model

$$q_{t}=k_{p}t^{\frac{1}{2}}+C,$$ (7)

where $\left(k_{p}\right)$ is the intra-particle diffusion rate constant and (C) is the intercept of the plot, which indicates the boundary layer effect.

• The Byod equation

$$B_{t}=-4.977-\ln\left(1-F\right),$$ (8)

where (F) is the fraction of solute adsorbed at any time and equals (q_t_/q_o_), with (q_o_) representing the ratio between the adsorbed and adsorbent masses at infinite time.

### 3.3 Adsorption isotherm study

The adsorption isotherm study was carried out following the guidelines provided in the literature (Usman et al., 2022a; Luo et al., 2016) using Equations 9–11. Under the following experimental conditions, this isotherm study was performed under different concentrations of phosphate ions ranging from 1 to 13.1 mg/L, pH = 8, adsorption time = 90 min, adsorbent dosage = 3.5 g/L, and temperature = 25 C. When compared to the Langmuir and Freundlich isotherm models, Table 3 illustrates that the best-fitted isotherm model for the experimental data is Langmuir. This was because the R^2^ value of the Langmuir isotherm model (0.9926) was lower than the R^2^ value of the Freundlich isotherm model (0.4207). Furthermore, the separation factor (R_L_) in the Langmuir isotherm (0.152) guaranteed that the experimental data were well-suited for this model’s representation as its value was between 0 and 1 (Desta, 2013). Therefore, this adsorption system’s mechanism was a single layer. The constant K_L_ for Langmuir was 0.4248 L/mg, which was related to the energy of adsorption. The value of mean adsorption energy equals 10.31 kJ/mol using the Dubinin–Radushkevich isotherm, which is in the range between 8 kJ/mol and 16 kJ/mol. Therefore, it indicated that the adsorption was controlled by chemisorption through ion exchange between the phosphate ions and the adsorbent surface (DCI) (Rahman et al., 2022).

• Langmuir equation

$$\frac{1}{q_{e}}=\frac{1}{Q^{o}}+\left(\frac{1}{K.Q^{o}}\right)\left(\frac{1}{C_{e}}\right).$$ (9)

• Freundlich equation

$$\log q_{e}=\log K_{F}+\frac{1}{n}\log C_{e}.$$ (10)

• Dubinin–Radushkevich equation

$$\ln q_{e}=\ln q_{\max}-BR^{2}T^{2}{\ln\left(1+\frac{1}{C_{e}}\right)}^{2},$$ (11)

where (q_e_) is the ratio of the solute adsorbed to the adsorbent masses at equilibrium, (q_max_) is the maximum adsorbent capacity, (C_e_) is the phosphate ion concentration at equilibrium in (ppm), (C_o_) is the initial concentration in (ppm), (Q^o^) is the maximum monolayer adsorption capacity, (K) is the Langmuir constant, which indicates the adsorption energy, (R_L_) is the separation factor, which equals $\frac{1}{1+K\;C_{o\;}}$ , (*K*_*F*_) is adsorption capacity (L/mg), (1/*n*) is the adsorption intensity, (R) is the universal gas constant, (B) is the Dubinin–Radushkevich constant, (T) is the temperature in Kelvin, and (E) is the mean adsorption energy, where ( $E=\frac{1}{\sqrt{2\;B}}).$

**TABLE 3:** Adsorption isotherm study results.

Isotherm model	Parameter	Value
Langmuir	R^2^	0.9926
	q_max_ (mg/g)	0.283
	K_L_ (L/mg)	0.425
	R_L_ (Separation factor)	0.152
Freundlich	R^2^	0.4207
	n	41.15
	K_F_ (L/mg)	0.264
Dubinin–Radushkevich	β (mol^2^/kJ^2^)	0.004
	E (kJ/mol)	10.31
	q_max_ (mg/g)	0.28

### 3.4 Thermodynamic study

Thermodynamic calculations can be used to describe whether the adsorption process occurs spontaneously or non-spontaneously and determine the driving force of the adsorption, which may be examined using the thermodynamic parameters of entropy change (ΔS), enthalpy change (ΔH), and Gibbs free energy change (ΔG) (Elsawy et al., 2022). Equations 12–15 could be used for the calculation of (ΔG), (ΔH), and (ΔS) (Elsawy et al., 2022):

ΔG=−RT lnk, (12)

$$k=\frac{C_{s}}{C_{eq}},$$ (13)

$$lnK=\frac{\Delta S}{R}-\;\frac{\Delta H}{RT},$$ (14)

ΔG=ΔH−TΔS, (15)

where T is the temperature in Kelvin, R is the universal gas constant, which equals 8.314 J/mol.K, (ΔS) is the entropy change of adsorption (kJ/mol.K), ΔH is the enthalpy change of adsorption (kJ/mol), ΔG is the Gibbs free energy change of adsorption (kJ/mol.K), C_eq_ is the equilibrium adsorbate concentration in solution (mg/L), C_s_ is the equilibrium adsorbate concentration in the solid adsorbent in (mg/L), and k is the equilibrium constant. In this research, thermodynamic study experiments were performed under following conditions: pH = 8, 3.5 g/L DCI solid waste adsorbent, 90 min adsorption time, different concentrations (1 mg/L, 5.5 mg/L, and 10 mg/L), and different temperatures (318 K, 328 K, and 298 K), as shown in Table 4.

**TABLE 4:** Important results of thermodynamic study.

Temperature change effect on phosphate ion adsorption					
Temperature (K)	Concentration of PO_4_^3-^ion (mg/L)	Ceq (mg/L)	Cs (mg/L)	k	ln k
298	1	0.14	0.86	6.14	1.82
	5.5	4.54	0.96	0.21	−1.55
	10	9.0900	0.910	0.100	−2.30
318	1	0.12	0.88	7.33	1.99
	5.5	2.850	2.650	0.93	−0.07
	10	8.9200	1.080	0.12	−2.11
328	1	0.09	0.91	10.11	2.31
	5.5	2.2	3.3	1.50	0.41
	10	8.5	1.5	0.18	−1.73

Thermodynamic parameters (∆H, ∆S, and ∆G) for phosphate ion adsorption						
Concentration of PO_4_^3-^ion (mg/L)	ln k	Temperature (K)	1/T (K^−1^)	ΔH (kJ/mol)	ΔS (kJ/mol.K)	ΔG (kJ/mol)
1	1.82	298	0.00336	115.00	0.39	−0.72
	1.99	318	0.00314			−8.49
	2.31	328	0.00305			−12.37
5.5	−1.55	298	0.00336	106.26	0.36	−2.13
	−0.07	318	0.00314			−9.40
	0.41	328	0.00305			−13.04
10	−2.30	298	0.00336	103.99	0.37	−6.23
	−2.11	318	0.00314			−13.62
	−1.73	328	0.00305			−17.32

**FIGURE 5:**
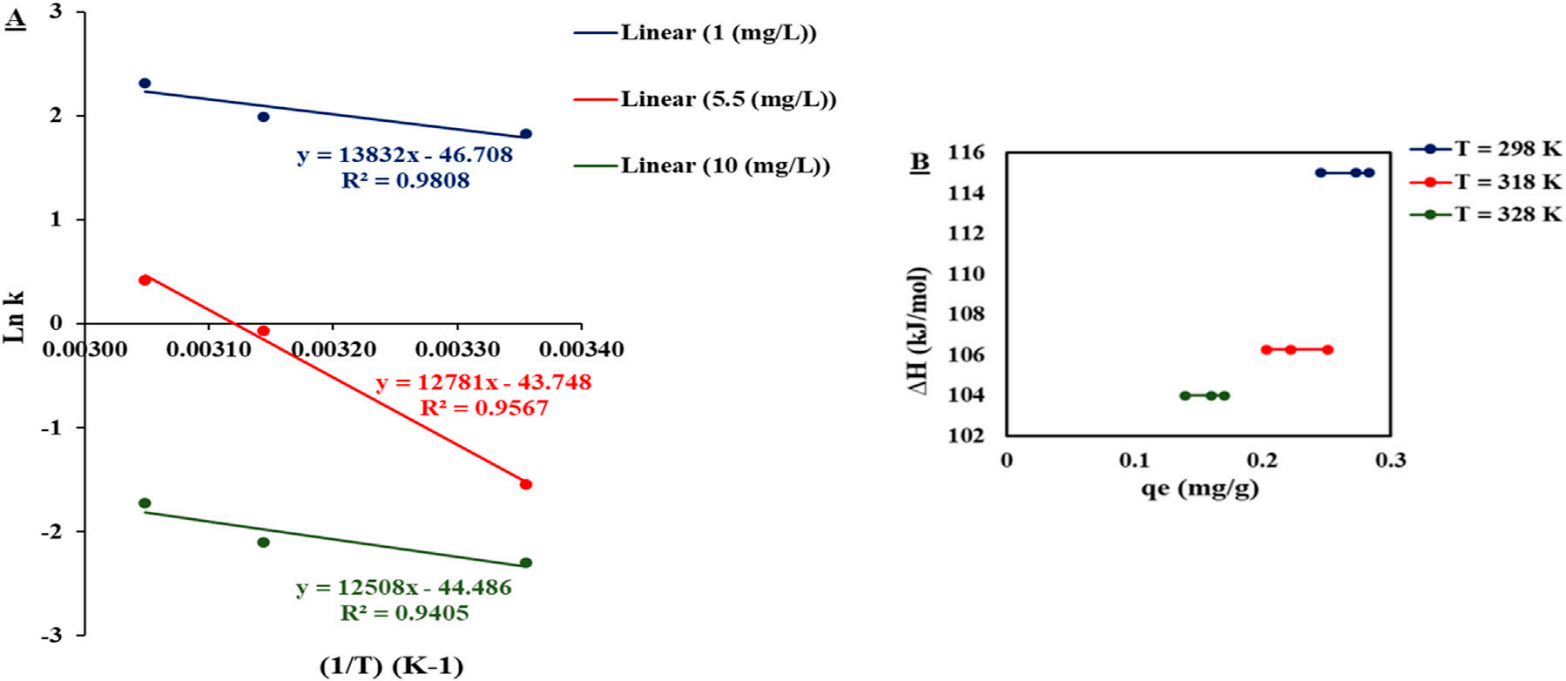
**(A)** Plot of (1/T) on the *x*-axis versus ln (k) on the *y*-axis. **(B)** Plot of q_e_ on the *x*-axis versus ∆H on the *y*-axis.

By plotting the values of “1/T” on the *x*-axis versus “ln k” on the *y*-axis, the value of ΔH could be determined from the slope, while the ΔS value could be determined from the intercept, as shown in Figure 5A.

The value of ΔH indicated the adsorbent surface heterogeneity via plotting of ΔH versus q_e_, as represented in Figure 5B, where the values of enthalpy at different temperatures (298 K, 318 K, and 328 K) were constant along different levels of q_e_, indicating that adsorption heat was not affected by the sites that existed on the DCI adsorbent surface. Thus, the adsorption mechanism was a single layer where all adsorption sites were equal in binding affinity. This conclusion was consistent with the isotherm study, which illustrated that the adsorption experimental results fitted with the Langmuir model, indicating the single-layer adsorption mechanism.

As observed in Table 4, the negative ΔG values indicated the spontaneous adsorption process. The positive values of ΔH indicated that the adsorption of phosphate ions from aqueous solutions by the DCI solid waste was an endothermic process. Endothermic adsorption occurs when the bond between the adsorbent and adsorbate is weaker than that between the adsorbent and solution and between the adsorbate and solution phase interactions (Silva and Baltrusaitis, 2020). The high enthalpy change values >100 kJ/mol indicated that the adsorption mechanism was chemisorption involving ionic bonds (Silva and Baltrusaitis, 2020).

### 3.5 Modeling of phosphate ion’s adsorption process

**FIGURE 6:**
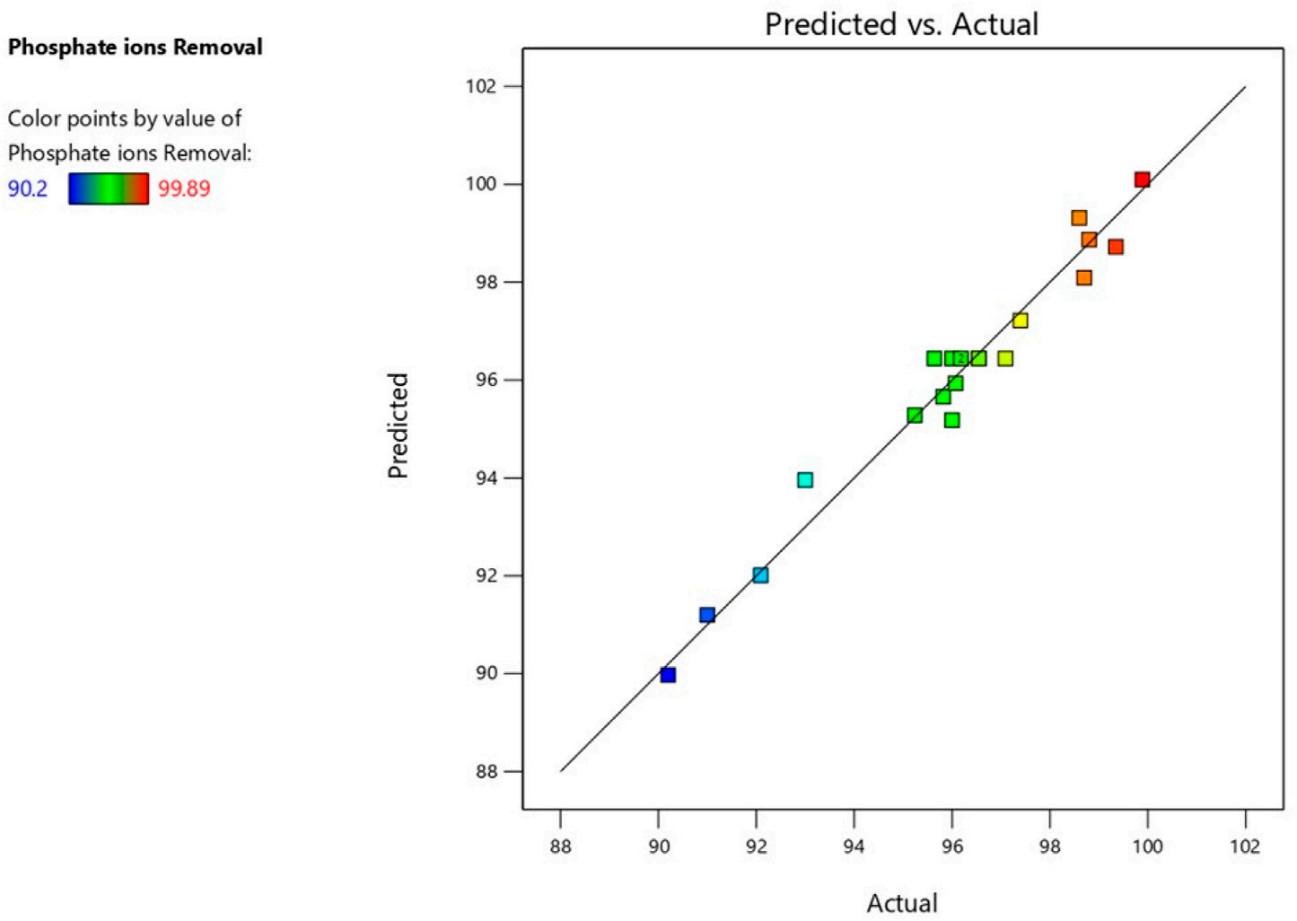
Phosphate ion removal predicted versus experimental results.

By using the experimental runs, the percentage of phosphate ions removed was calculated under the conditions presented in Table 5. Models reflecting the relationship between the parameters of the process and its response were produced using Design Expert V13. P and F values were calculated using the analysis of variance (ANOVA) approach at a 95% confidence level to determine whether the generated models were significant and appropriate. The quadratic model was the most significant model for phosphate removal. Equation 16 provided an example of the module, and Table 5 presents the findings of the ANOVA study. Additionally, all R-squared values supported the reasonable agreement between the estimated and experimental results for phosphate ion elimination, as supported by Figure 6. This accord attested to the models’ suitability.

$$X=98.24+0.02\;A+0.049\;B-1.36\;C-0.06\;AC-{0.0003\;B}^{2},$$ (16)

where C is the amount of the adsorbent (g/L), B is the contact time (min), and A is the concentration of phosphate ions (mg/L).

**TABLE 5:** ANOVA results for the process of phosphate ion removal.

Source	F-value	*p*-value
Model	54.91	<0.0001
A	25.56	0.0002
B	8.06	0.0131
C	229.17	<0.0001
AC	10.92	0.0052
B^2^	5.52	0.0340
R^2^	0.9515	
Adjusted R^2^	0.9342	
Predicted R^2^	0.8776	

#### 3.5.1 Variation in phosphate ion removal percentage with experimental factors

**FIGURE 7:**
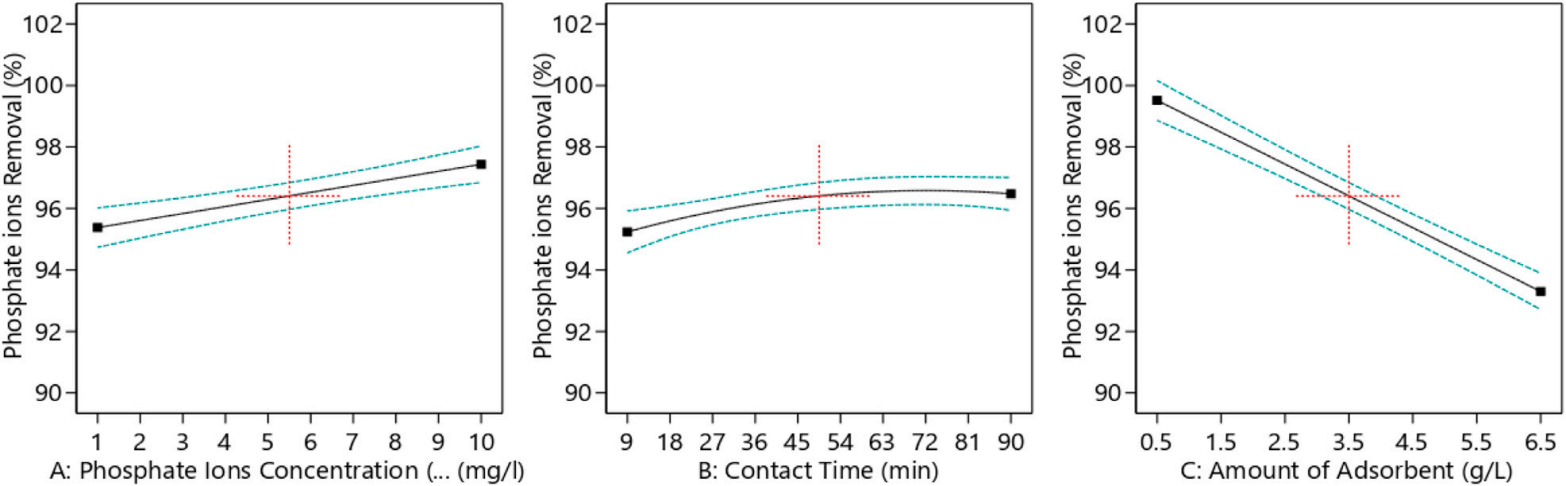
Phosphate removal percentage versus each process parameter. **(A)** Phosphate ion concentration, **(B)** contact time, and **(C)** amount of the adsorbent.

Figure 7 demonstrates the effect of each process parameter on the phosphate removal percentage. The amount of adsorbent had the greatest impact on the phosphate removal percentage as it increased the driving force and reduced the mass transfer resistance, which increased the process efficiency and, subsequently, the removal percentage. On the other hand, contact time initially had the lowest effect on the phosphate ion removal from aqueous solutions compared to the other factors, according to the results of ANOVA. Increasing the adsorbent dose initially led to an increase in the phosphate ion removal percentage; however, beyond a certain point, additional doses of the adsorbent caused particle agglomeration, which reduced specific surface area and ultimately decreased the effectiveness of phosphate ion removal.

#### 3.5.2 Interaction of process parameters with dye removal percentage

**FIGURE 8:**
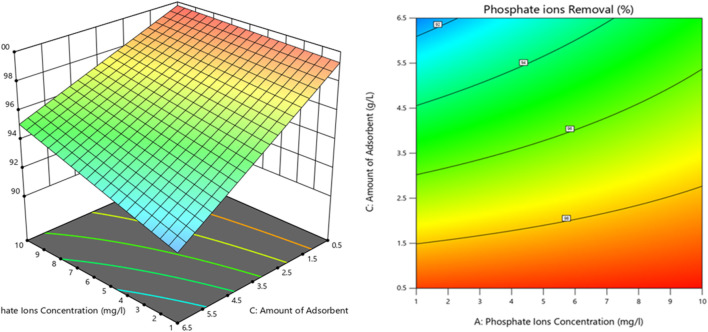
3D plot and contour map connecting the interactions of two process variables and phosphate removal efficiency.

Figure 8 shows the relationship between the dye removal percentage, phosphate ion concentration, and adsorbent amount interaction (AC) through 3D and contour plots.

### 3.6 Process optimization

To obtain the optimal values for the three process-independent factors (phosphate ion concentration, adsorbent amount, and adsorption time) that had an impact on the percentage of phosphate ion removal (which is the dependent process response), numerical optimization was performed using the Design Expert V13 program. This process involved combining the desirability of all independent variables into a single value to find the best response goals.

The objective of the optimum process was to maximize the concentration of phosphate ions to remove as many pollutants as possible in addition to minimizing both contact or adsorption time and the adsorbent’s amount to decrease the cost of the process. Fifty feasible choices with differing degrees of desirability were generated using Design Expert V13, and the optimum phosphate removal efficiency obtained was 98.9% under 10 mg/L initial phosphate ion concentration, 10 min adsorption time, and 0.5 g/L adsorbent amount.

### 3.7 Desorption and DCI adsorbent reusability

Salts, acids, and bases could be used to desorb phosphate (Usman et al., 2022c). When phosphate and the adsorbent interact strongly, salts are not highly effective (Usman et al., 2022c). Acids and bases were more effective in such cases (Almanassra et al., 2021). In most cases, sodium hydroxide was used to desorb phosphate. Using sodium hydroxide as a stripping agent/desorption solution, the active sites on the adsorbent surface could be regenerated after the sample had been separated from the mixed solution in batch adsorption experiments (Trinh et al., 2020). The sample was dried before use in the next batch study. Due to the strong influence of -OH on phosphate adsorption, sodium hydroxide was the best regenerating agent (Jia et al., 2020). The regeneration efficiency increased as sodium hydroxide concentration increased (Jia et al., 2020). In this study, DCI solid waste was regenerated and desorbed in 10 mg/L phosphate solutions at pH = 8, adsorbent amount = 3.5 g/L, and temperature = 25 °C. The DCI-adsorbed 10 mg/L phosphate ion solution was washed with 0.1 M NaOH for 3 h. This mixture was then filtered and dried at 60°C for 4 h to obtain regenerated DCI. A 10 mg/L solution of phosphate ions was again adsorbed onto the regenerated DCI at pH = 8, adsorbent amount = 3.5 g/L, and temperature = 25 °C. Four consecutive cycles of adsorption and desorption were then performed to investigate the reusability of DCI solid waste as an adsorbent.

**FIGURE 9:**
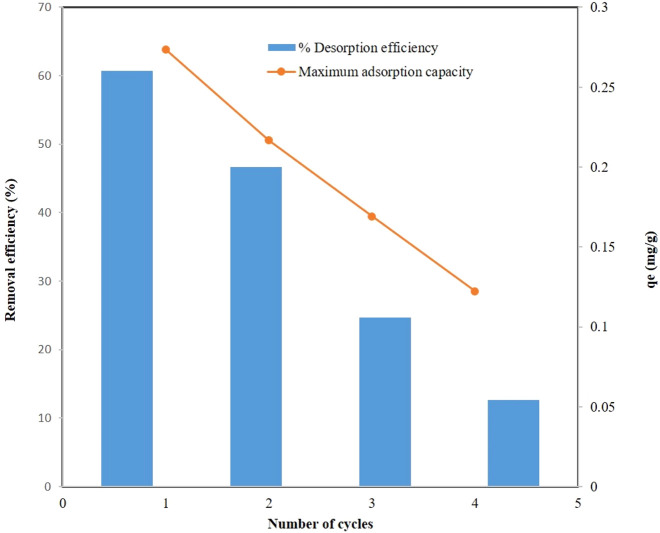
Desorption results of phosphate ions from the DCI solid waste.

A decrease in adsorption capacity (q_e_) could be observed in Figure 9 with the increasing number of cycles. Adsorption capacity decreased, suggesting that phosphate ions bound strongly to some adsorption sites and were difficult to elute using 0.1 M NaOH during desorption. DCI adsorption capacity declined continuously as the number of cycles increased, and the non-renewable sites increased accordingly. After four cycles, it could be observed that a large fraction of the adsorption capacity was lost. Accordingly, DCI solid waste was an adsorbent with good reusability and adsorption capacity up to the fourth cycle.

## 4 Comparison between the DCI solid waste as an adsorbent and the other adsorbents

Based on adsorption capacity and operating conditions, the results of the present study were compared with those from similar studies. As shown in Table 6, several adsorbents have been reported to be capable of eliminating phosphate ions from aqueous solutions. When compared with other previously reported adsorbents, the DCI solid waste could be a good adsorbent for adsorbing phosphate ions from aqueous solutions. The DCI solid waste consisted mainly of magnesium oxide (MgO) along with other oxides acting as impurities, which decreased its ability of phosphate ion adsorption in high capacity. Therefore, modification of the DCI solid waste might increase its adsorption capacity toward phosphates from aqueous solutions under different experimental operating conditions. It was observed in Table 6 that the modified adsorbents, such as activated carbon impregnated with iron (AC impregnated with Fe) and magnesium–iron-layered double hydroxides (Mg–Fe-LDH), demonstrated the greatest adsorption capacities of 14.1 mg/g and 28.3 mg/g respectively, compared to other adsorbents. As represented in Table 6, the adsorbent dose, initial concentration of phosphate ions, and the contact time in the case of adsorption using the DCI solid waste were lower than those of most of the other adsorbents, which made its adsorption capacity low compared with them. Moreover, the DCI solid waste was generated (hundred thousand tons/year) from foundries and was used in this research as an adsorbent for phosphate ions from an aqueous solution. This approach makes it more economical and environmentally sustainable compared to other documented adsorbents. Additionally, its regeneration was easy to perform compared to the adsorbents documented in Table 6.

**TABLE 6:** Comparison between the DCI solid waste and other adsorbents for the removal of phosphate ion.

Adsorbent	pH	Contact time	Initial concentration (mg/L)	Adsorbent dose (g/L)	Maximum adsorption capacity (q_max_) (mg/g)	Temperature (^o^C)	Reference
Zeolite	7	24 h	5–2,000	20	0.71	25	Wang et al. (2016b)
Modified bentonites	6.5	60 min	25	20	8.33	25	El-Maghrabi et al. (2022)
Dolomite	7.6	20 min	10	20	0.17	25	Chen et al. (2013)
Graphene oxide (GO)	6	6 h	0.1–0.5	0.05	1.68	25	Almanassra et al. (2021)
ZnCl_2_-activated coir pith carbon	7	2 h	10–40	12	5.1	35	Luo et al. (2015)
Ceramic	4–10	30 min	10–100	10	5.96	25	Wang et al. (2016b)
AC impregnated with Fe	3.8	3 h	11.82–42.96	2	14.12	25	Luo et al. (2016)
Mg–Fe-LDH	5	300 min	20–800	3.3	28.3	25	Xu et al. (2020)
DCI solid waste	8	90 min	1–13.1	3.5	0.28	25	This study

## 5 Conclusion

Water scarcity is one of the most urgent challenges faced globally. The present study aims to solve the issue. This method entails utilizing ductile cast iron hazardous solid waste as an adsorbent for phosphate ions in wastewater, thus converting waste into a valuable resource by facilitating its utilization in water treatment. For the DCI solid waste, characterization was carried out via XRD, FTIR, PSD, and XRF. The adsorption type and mechanism were determined by the kinetics and isotherm studies. The chemisorption adsorption mechanism was proved through the fitting of the PSO kinetics model with the experimental results in combination with the results from XRD and FTIR analyses. The adsorption system was a single-layer system with a maximum adsorption capacity of 0.28 mg/g. The Dubinin–Radushkevich model suggested that the adsorption was based on the ion exchange between phosphate ions and the surface of the DCI solid waste due to the value of activation energy (E = 10.61 kJ/mol). Thermodynamic study illustrated the endothermic nature of this adsorption system based on the highly positive values of enthalpies (∆H) > 100 kJ/mol at different temperatures of 298 K, 318 K, and 328 K. Using the Design Expert software program, the optimal experimental conditions of pH = 8, temperature = 25°C, contact time = 9 min, phosphate ion concentration = 10 mg/L, and adsorbent amount = 0.5 g/L were found to yield the best removal efficiency for phosphate ions (98.9%) from aqueous solutions.

## Data Availability

The original contributions presented in the study are included in the article/supplementary material; further inquiries can be directed to the corresponding authors.
